# A Matching Algorithm for Underwater Acoustic and Optical Images Based on Image Attribute Transfer and Local Features

**DOI:** 10.3390/s21217043

**Published:** 2021-10-24

**Authors:** Xiaoteng Zhou, Changli Yu, Xin Yuan, Citong Luo

**Affiliations:** School of Ocean Engineering, Harbin Institute of Technology, Weihai 264209, China; zhouxiaoteng@stu.hit.edu.cn (X.Z.); xin.yuan@upm.es (X.Y.); luocitong@gmail.com (C.L.)

**Keywords:** sensor data fusion, multimodal image matching, attribute transfer, image processing

## Abstract

In the field of underwater vision, image matching between the main two sensors (sonar and optical camera) has always been a challenging problem. The independent imaging mechanism of the two determines the modalities of the image, and the local features of the images under various modalities are significantly different, which makes the general matching method based on the optical image invalid. In order to make full use of underwater acoustic and optical images, and promote the development of multisensor information fusion (MSIF) technology, this letter proposes to apply an image attribute transfer algorithm and advanced local feature descriptor to solve the problem of underwater acousto-optic image matching. We utilize real and simulated underwater images for testing; experimental results show that our proposed method could effectively preprocess these multimodal images to obtain an accurate matching result, thus providing a new solution for the underwater multisensor image matching task.

## 1. Introduction

In recent years, many organizations have gradually begun to obtain resources such as oil and gas from the deep sea to meet the needs of human and industrial sustainable development. With the gradual deepening of the development of marine resources, deep-sea exploration activities will become more frequent. Current mainstream deep-submersibles, such as autonomous underwater vehicles (AUVs) and remotely operated vehicles (ROVs) are also equipped with more advanced acoustic and optical sensors. These sensors have played an outstanding role in seabed geomorphological mapping, target recognition and classification, biological research, resource exploration, environmental monitoring, and other fields [1,2,3,4,5,6]. Sonar is the most commonly used sensor in the field of deep-water exploration, which could collect images of marine targets at a relatively long distance and is not disturbed by turbidity. However, it encounters special cases in the imaging process, such as low signal-to-noise ratio (SNR), low resolution, and low feature repeatability [6]. In addition, there are problems such as blurred target edges, serious distortion and poor overall image quality in imaging. What is worse is when sonar detects the same area from different angles; these areas may have dramatic lighting changes [7]. Optical cameras could intuitively provide high-resolution and highly recognizable target images, but due to the scattering effect of light in seawater and the influence of the medium on its absorption, most of the optical images are blue-green and the imaging distance is severely limited. Even if the acoustic and optical sensors are in the same scene, there are significant differences between the two in terms of clarity, color, dynamic range, signal-to-noise ratio, and structural similarity. In order to alleviate their respective limitations, the advantages of the two should be combined. Acousto-optic fusion technology has developed rapidly in recent years; the technology is active in marine observation and joint positioning applications. For example, optical image information (color, contour, etc.) could be used to further explore the details of target objects in acoustic images [8] or using sonar and optical camera to achieve target tracking in turbid waters.

A basic research work to promote acousto-optic fusion technology is to realize the local feature matching of underwater multimodal images. However, the traditional matching ideas based on extracting regional local features such as points, lines, and contours, as described by [9,10,11], usually require handmade features and are easily affected by external changes; most methods were originally developed for optical images, so that the performance on acoustic images is not satisfactory. Some researchers have tried using traditional descriptors [6,7,12,13] to process underwater images. However, these methods are usually only for a specific sonar research application and do not have generalization. Moreover, the image processing is more cumbersome and requires rich professional experience to intervene in the processing. It is difficult to form an effective and fixed processing method. Although the semantic structure of underwater acousto-optic image pairs is similar, there are great differences in texture features and noise distribution. In a complex underwater environment, the imaging of acousto-optic sensor will change with the angle and scale, and the subsequent affine problem has also become the difficulty of the underwater matching task. Committed to achieving the underwater acousto-optic image matching task with as little human intervention as possible, our method is mainly divided into two steps:

First, use the image attribute transfer algorithm to eliminate the difference between the acoustic and optical images. Second, a more expressive local feature descriptor based on deep learning is introduced to improve the accuracy and robustness of matching.

Additionally, in view of the difficulty and high cost of underwater acousto-optic data acquisition, in order to better meet the needs of experiments and further optimization, this paper adopts the method proposed in [14] to complete image simulation; the purpose is to generate realistic underwater acousto-optic images to expand testing samples. Final experimental results show that our proposal could quickly and accurately achieve feature point detection and local feature matching on acousto-optic images with less human intervention and it could be applied to a variety of underwater scenes.

The main framework of the paper follows. Section 2 reviews classic and latest research regarding underwater image matching. Section 3 presents the detailed method of our work. Section 4 demonstrates the experiment details. Section 5 verifies the algorithm and compares the evaluation results. Section 6 provides the discussion and outlook. The conclusion is drawn in Section 7.

## 2. Related Work

The research inspiration of underwater acoustic and optical image matching is based on multimodal image feature matching. Image matching is a classic research topic in the field of computer vision. It is mainly divided into region and local feature matching. Region matching focuses on the correlation within a group of comparative image sets [8]. Local feature matching focuses on the details of the target object, such as point, line, or contour, and it could support a wider range of applications in underwater tasks.

In 2004, Fusiello and Murino [15] earlier proposed a joint acousto-optic device used in underwater environment perception, and underwater scene modeling issues verify the effectiveness of the proposed ideas. Negahdaripour [16] introduced in more detail the system calibration and three-dimensional object reconstruction task, acousto-optic reveal sensor fusion technology advantages, and introduced a new method of photoacoustic stereo calibration. In the medical field, some researchers have also completed much research on acousto-optic information fusion technology [17].

High quality image acquisition is the foundation of acousto-optic fusion technology research. For this, extensive research on improving and restoring underwater optical images has been carried out [18,19,20]. In [21,22], the restoration and feature extraction methods of sonar images have been studied. These are some attempts at underwater image preprocessing.

In the field involving underwater sonar matching, Vandrish [7] compared the results of side scan sonar (SSS) image matching using scale-invariant feature transform (SIFT) and other traditional local feature descriptors, and concluded that SIFT performed best among the traditional matching methods. The SIFT registration algorithm for two synthetic aperture sonar images was studied in [12], and two ideal sonar track image registration geometric models were proposed. Kim [13] proposed an idea of associating the detected key points with Harris corner detection in a general sonar image registration task. Hurtós [21] used features based on Fourier transform to match forward-looking sonar images and achieved satisfactory results, but this method was limited by the rotation and translation range. Toro [23] tried to use convolutional neural network (CNN) to learn the match mapping of sonar images, and proposed an algorithm to generate matching pairs for training from the labeled target. This method could directly learn the matching function from the labeled data without any manual feature engineering. The final result shows that the accuracy of sonar image matching is higher after the feature processing of CNN, while the accuracy of the classical keypoint method is lower. Pham [24] used the guidance of block matching, a segmented sonar image with a self-organizing map for registration and mosaic of SSS images. Yang [25] put forward a kind of image matching algorithm based on CNN, the purpose was to address the problem that traditional image feature representations and similarity assessment are not learned jointly. It can improve the matching accuracy of deep-sea sonar images in a dynamic background with low intensity and high noise scenes. This model could train sonar image texture features without any artificial design feature descriptor.

Previous studies in sonar image matching mainly focus on image processing of a single sensor; sonar image matching studies mainly serve for image registration [13,21]. In recent years, some researchers have begun to study how to combine other sensors (mainly optical sensors) on the basis of sonar technology for matching research.

Liu [8] tried to design a region-based filter to manage the problem of acousto-optic matching. This method could eliminate the impact of changes in the viewing angle and environmental changes during sensor imaging. At the same time, they proposed an iterative algorithm to enhance the image, and increase the proportion of effective information in the graph, and then use morphological filtering for noise suppression. Finally, Gaussian multiscale images were used to optimize the matching results to reduce scale errors. The experimental results show that this method could initially realize the macroscopic matching on the acousto-optical image area. However, this method requires the design of a variety of attached algorithms and filters, requires extensive expert experience and manual intervention, and the overall process is relatively complicated, and it is impossible to match the local feature details of the image. It does not have obvious advantages in matching accuracy, and there are some limitations in the research of improving the autonomous ability of deep submersibles.

Hyesu Jang [26] proposed a style transfer method based on CNN combined with traditional feature descriptors to match underwater acousto-optic images. The idea of this article was also inspired by their creativity, but their method can only finish the matching in one style, such as generating a sonar image in an optical style, and then finally match optical style. Secondly, the parameter setting of their style transfer method is not clear, and there are many custom parameters. Many attempts are required during the experiment. The images before and after the transfer cannot establish a deep mapping relationship; furthermore, during the process of image style transfer, the introduction of noise can easily destroy the original structure and detail of the image.

In this article, we propose a new method for matching underwater acoustic images and optical images. Based on the advanced image attribute transfer algorithm [27], we introduce a learned descriptor [28] with stronger expressiveness in complex matching tasks. The image preprocessing opens the way for subsequent matching and further strengthens the matching effect based on local features. The introduction of the learned descriptor provides a reference for the new method design of the underwater matching task.

## 3. Method

For image fusion tasks, the best spatial transformation must be found to make the points in the same spatial position in the two images correspond. In this section, we propose a new method for underwater acousto-optic image matching to further solve the problem of acquiring data in underwater engineering tasks.

The method has the following three aspects:

- Based on the analogy of acoustic and optical image attributes, it combines the advantages of CNN depth feature extraction to realize image visual attribute conversion, and then eliminates the differences between the acousto-optic images;
- To match the generated target image and the original image in the acoustic domain and the optical domain, respectively, using the current advanced learned descriptor;
- The data aggregation method is used to display the calibrated matching correspondence on the original acoustic and optical images.

**Figure 1:**
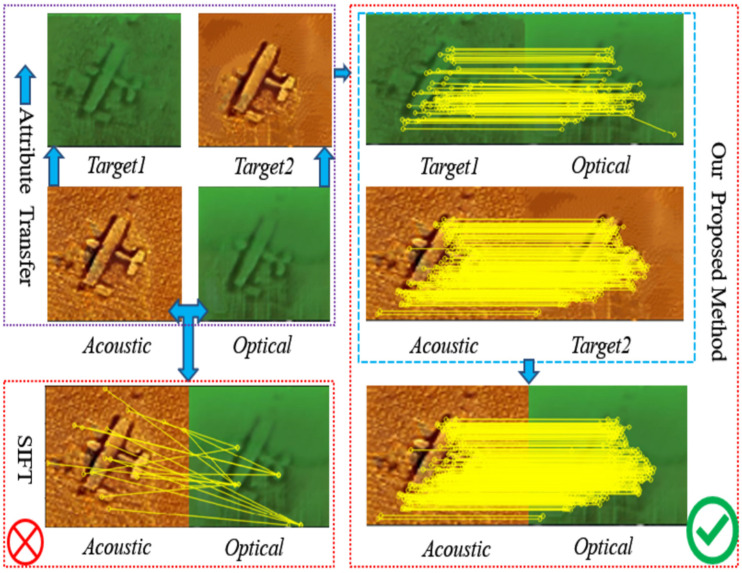
The process of the proposed method.

The process of the proposed method is depicted in Figure 1. First input an acoustic image and an optical image of one target, and then use the method of attribute transfer to obtain the corresponding target images of the two. Then match the acoustic image and optical image with the target image generated by the other party, finally eliminate false matches and map correspondences to the original image pair. The matching processes are carried out in acoustic domain and optical domain, respectively, in order to ensure the matching accuracy.

### 3.1. Image Attribute Transfer

The visual attribute transfer method introduced in [27] has been widely used in image texture, color, content, and style transfer scenes. The core of this method is to give full play to the superior performance of CNN’s deep feature extraction in the image processing procedure. The deep pyramid features extracted by a deep CNN are used to construct the semantic dense correspondence between image pair, and gradually adjust and optimize to achieve the attribute transfer of two images.

**Figure 2:**
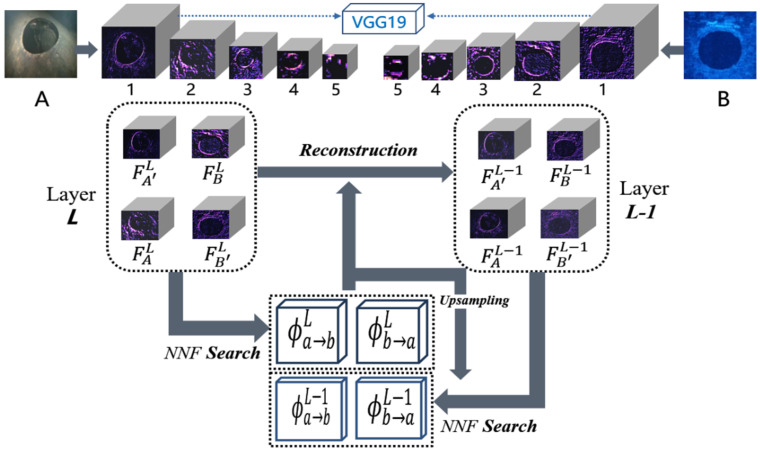
The image attribute transfer process.

As shown in Figure 2, we transmit sonar images and underwater optical images with relevant semantic information to the CNN network (VGG19 [29]) for the construction of a feature pyramid and select five groups of depth feature maps $\left\{F_{A}^{L}\right\}$ and $\left\{F_{B}^{L}\right\}$ (L=1…5). From the first layer to the fifth layer, image details are gradually lost, and almost only the high-level semantic information remains at the top layer. $F_{A}^{L}$ is a feature map which represents the response of image A on the scale L, and $F_{B}^{L}$ has a similar definition in reverse direction, corresponding $F_{A^{\prime }}^{L}$ indicates that, when reconstructed the image on scale *L*, the feature map has the content structure of image A and style details of image B. After establishing the mapping relationship at the coarsest layer (*L* = *5*), the next step is to iterate from the high level to the low level. Its application in the field of underwater acoustic image and optical image fusion is described as follows:

Initially, the features of A′ and B′ are unknown. We estimate these from coarse to fine, which requires good initialization at the coarsest layer (L=5). Assume $F_{A^{\prime }}^{L}=F_{A}^{L}$ and $F_{B^{\prime }}^{L}=F_{B}^{L}$ are satisfied in the coarsest layer. At this time, it is equivalent to ignoring the detailed information of the image by default, and the semantic information is consistent.

**Figure 3:**
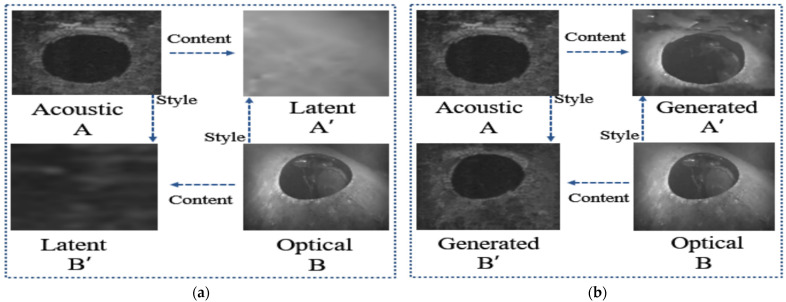
Visualization of underwater acousto-optic image attribute transfer: (**a**) the raw acoustic and optical image pair before image attribute transfer; (**b**) the acoustic and optical image pair after image attribute transfer.

As shown in Figure 3, we decompose the acoustic image and the optical image into two domains of style and content synchronously, and then define the image with style features as the latent image, and fill in the content on it. Under the premise of ensuring the stability of the target content structure, we gradually unify the colors, textures, and styles of areas with similar content structures. The process description is mainly divided into feature alignment and image reconstruction.

#### 3.1.1. Feature Alignment

Before image reconstruction, it is necessary to establish a position mapping between feature maps, that is, feature alignment. This method mainly adopts nearest neighbor field (NNF) for feature alignment, in which the algorithm used to calculate the approximate NNF between two images is well applied in [30]. In this study, the similarity between patches is mainly considered and we use mapping functions $\phi^{L}{}_{a\to b}$ to represent a forward NNF we estimated and $\phi^{L}{}_{b\to a}$ represents a reverse NNF. In other words, $\phi^{L}{}_{a\to b}$ means to search the patch p in $F_{A}^{L}$ and find another patch q with the smallest distance from it in $F_{B}^{L}$. $\phi^{L}{}_{b\to a}$ is similarly defined in the reverse direction. The reference formula follows:

(1) $$\phi_{a\to b}^{L}\left(p\right)=\arg_{q}\min\sum_{x\in N\left(p\right),y\in N\left(q\right)}\left({\|{\bar{F}}_{A}^{L}\left(x\right)-{\bar{F}}_{B^{\prime }}^{L}\left(y\right)\|}^{2}+{\|{\bar{F}}_{A^{\prime }}^{L}\left(x\right)-{\bar{F}}_{B}^{L}\left(y\right)\|}^{2}\right)$$

where N(p) is the patch around p and *F*(*x*) is a vector in position x. ${\bar{F}}^{L}\left(x\right)$ represents normalized features in patch similarity metric, which are better for matching.

Equation (1) could easily obtain four variables by using the ideal assumption conditions at the coarsest layer, and then obtain the analogy mapping relationship between the acousto-optic images at layer L=5. After that, this analogy mapping relation is used to infer the variables between adjacent lower layers.

#### 3.1.2. Image Reconstruction

The intention of image reconstruction is that the ideal latent image $A^{\prime }$ fully retains the content structure of the original sonar image A while referring to the style details of the optical image B. The ideal latent image $B^{\prime }$ has a similar explanation in the reverse direction; this idea is vividly illustrated in [31].

After feature processing is completed, to reversely infer the lower layer from the coarsest layer of the image feature layers, until the first layer gets $F_{A^{\prime }}^{1}$ and $F_{B^{\prime }}^{1}$, that is, the final target images are generated. In brief, based on the known mapping relationship of layer L, the mapping relationship of the layer *L* − 1 is gradually explored.

**Figure 4:**
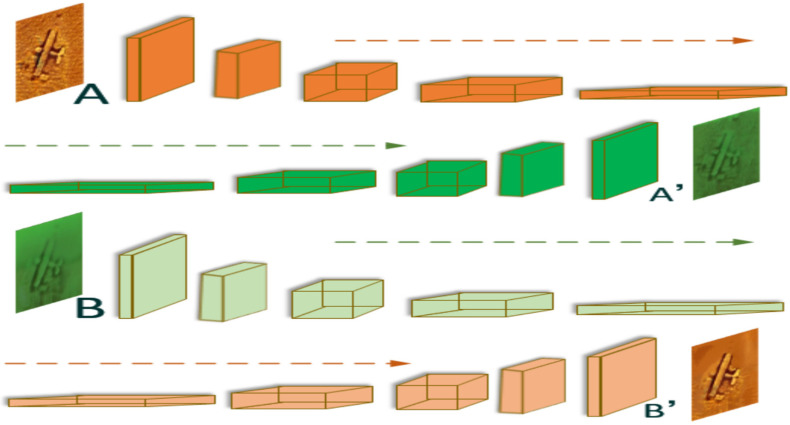
The image reconstruction process.

As shown in Figure 4, taking $A^{\prime }$ generated by A and *B* as an example, based on the ideal assumptions of the coarsest layer ($F_{A^{\prime }}^{L}=F_{A}^{L}$), deconvolution of $A^{\prime }$ is carried out by using the feature map of layer  of A($F_{A}^{L}$). To satisfy $A^{\prime }$ maintaining the content structure of A while incorporating the style details of B, the deconvolution result of $F_{A}^{L}$ needs to be adjusted before input to $F_{A\prime }^{L-1}$. In this adjustment, a custom weight coefficient W is introduced to control the similarity of $A^{\prime }$ to A and B; that is, W weights the proportion of content and style, and is modified with the change of layer number *L*. Formulas follow:

(2) $$F_{A\prime }^{L-1}=F_{A}^{L-1}\circ W_{A}^{L-1}+R_{B}^{L-1}\circ \left(1-W_{A}^{L-1}\right)$$

(3) $$F_{B^{\prime }}^{L-1}=F_{B}^{L-1}\circ W_{B}^{L-1}+R_{A}^{L-1}\circ \left(1-W_{B}^{L-1}\right)$$

By using Equation (2) for analysis, $R_{B}^{L-1}$ is obtained by deconvolution of $R_{B}^{L}$, and $R_{B}^{L}$ is obtained by deformation of $F_{B}^{L}$ in order to be close to $F_{A}^{L}$ in the content structure. The deformation criterion is based on the double constraint $\left(\phi_{a\to b},\phi_{b\to a}\right)$ of $F_{A}^{L}$ and $F_{B}^{L}$, and matching is carried out according to the search result of NNF. Equation (3) is similarly defined in reverse direction. The finally obtained $F_{A}^{L-1}$ and $F_{B}^{L-1}$ are combined with the feature of *A* and B at layer L−1, where ° is element-level multiplication. After obtaining the four variables of layer L−1: $F_{A}^{L-1}$, $F_{A^{\prime }}^{L-1}$, $F_{B}^{L-1}$, and $F_{B^{\prime }}^{L-1}$, the mapping relationship between images of this layer can be solved. Similarly, using this iterative method, we could finally get target images $A^{\prime }$ and $B^{\prime }$. It should be noted that we input two types of images and output two types of images. Using this method to establish the pixel location mapping relationship, the image $A^{\prime }$ is based on the content of the sonar image and the style is referenced to the optical image, while image $B^{\prime }$ is on the opposite side.

### 3.2. Learned Descriptor

**Figure 5:**
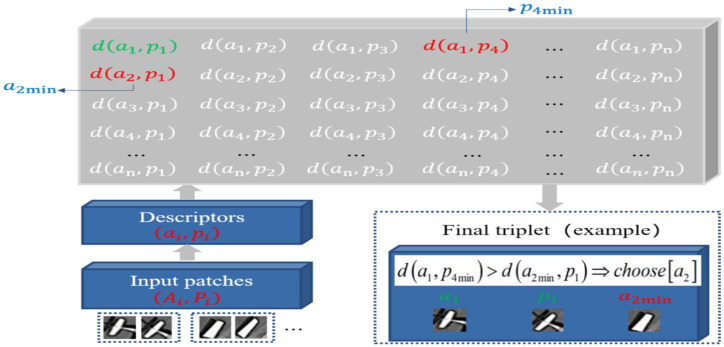
Schematic diagram of the HardNet sampling procedure.

In view of the self-limitations of the underwater acousto-optic image proposed above, we tried to introduce the current advanced learned descriptor HardNet to solve this challenging matching task. The design of HardNet is inspired by the Lowe’s matching criterion for SIFT. Many experiments show that its performance in matching task is much better than traditional local feature descriptors and other learned descriptors [28]. The sampling procedure and distance constraints of input patches are shown in Figure 5.

One training batch contains two batches and n matching pairs, each of which could be represented by $\left(a_{i},p_{i}\right)$, respectively. The $L_{2}$ distance matrix follows:

(4) D=cdist(a,p)

(5) $$d(a_{i},p_{j})=\sqrt{2-2a_{i}p_{j}},i=1\ldots n,j=1\ldots n$$

**Figure 6:**
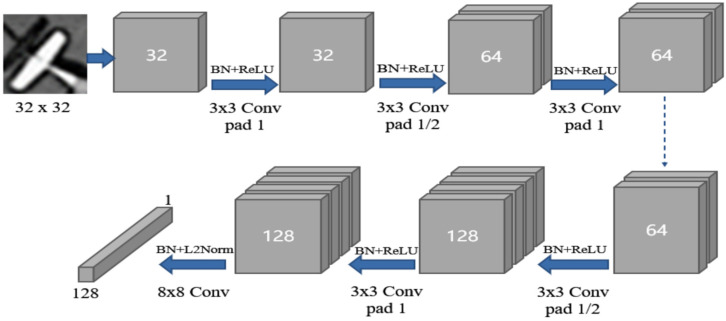
The network architecture of HardNet.

HardNet introduces a new loss for metric learning based on the CNN structure of L2-Net [32], and has been trained on Brown and HPatches datasets. When designing the network, every convolutional layer is followed by batch normalization (BN) and the activation function ReLU, except for the last one. Dropout regularization strategy is used before the last convolutional layer. The specific network structure is shown in Figure 6.

The design of the acousto-optic matching model adopts an end-to-end idea. In the preprocessing stage, we use the VGG19 model with better generalization performance. The process principle of the underwater acoustic and optical image matching could be expressed as the following Algorithm 1.

**Algorithm 1.** Underwater acoustic and optical image matching algorithm (UAOM)

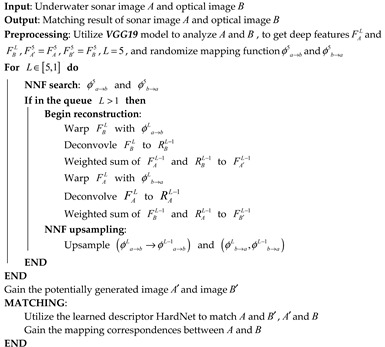



## 4. Experiment

### 4.1. Test Data Sets

**Figure 7:**
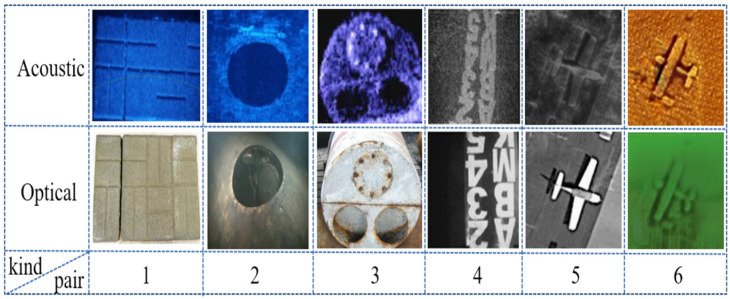
The test samples set: (**1**) paver bricks, affine transformation; (**2**) a hatch, affine transformation; (**3**) a mine shape, affine transformation; (**4**) a letter string, affine transformation; (**5**) plane, DIDSON style; (**6**) plane, SSS and underwater optical style.

In order to verify the effectiveness of the method in this paper, we selected 6 groups of image pairs for the test experiment, as shown in Figure 7.

Image pairs 1 and 2 are paver bricks and a hatch of one sunken plane, respectively, in which their acoustic images were captured by the ARIS Explorer 3000, which is dual frequency identification sonar (DIDSON); all the original images were provided by (SOUND METRICS, Washington, WA, USA). [33].

Image pair 3 is a mine shape and image pair 4 is a letter string; both their acoustic images were obtained by DIDSON, which were derived from [34] and [26], respectively.

Image pair 5 was generated using the style transfer method proposed in [14], in which the style of the acoustic image was transferred by DIDSON, and the optical image is real.

Image pair 6 was also generated using the method proposed in [14], in which the style of the acoustic image was transferred to the more common SSS, and the optical style was transferred to the mode of underwater optics. In order to increase contrast effect, the RGB mode is used to display.

### 4.2. Experimental Control Groups Sets

In terms of the control experiment, we introduce the classical image local-feature descriptors SIFT, SURF, and BRSIK [35] as the comparison, which, respectively, have excellent performance in robustness and speed, especially for underwater images with large blur. At the same time, in order to make the performance of HardNet descriptor more expressive, we introduce HesAffNet [36] as the detector to complete the matching task together.

## 5. Results and Evaluation

### 5.1. Evaluation Indexes Sets

We introduce the number of good matches (GM), average number of inliers (INL) per matched pair, matching accuracy (MA), and running time (RT) as the four indexes to evaluate match methods. GM measures the robustness, INL and MA measure the accuracy of the algorithm, and RT measures the real-time performance of the algorithm. For each image pair, we take the indexes average of ten test results as the final evaluation results.

1. GM: We adopt the number of good matches in per image pair when the ratio is 0.8 to measure the adaptability robustness of the method. The larger GM obtained for each group of images, the better the performance of the matching method.
2. INL: We take the average number of inliers in per image pair when the ratio is 0.8 to reflect the accuracy of the method, the higher the value, the better the performance.
3. MA: We introduce the matching accuracy to reflect the effective utilization of our algorithm; MA is numerically equal to the ratio of INL to GM. To a certain extent, MA could reflect the coordination between the detector and descriptor.
4. RT: In underwater engineering operations, real-time operation is a fixed requirement, so we introduce RT as the time evaluation index to measure the matching time, so as to verify the complexity of our algorithm.

### 5.2. Test Tools and Environment Details

All methods were implemented under the Windows 10 operating system using Python 3.7 with an Intel Core i7-9700 3.00 GHz processor, 16 GB of physical memory, and one NVIDIA GeForce RTX2070s graphics card. SIFT, SURF, and BRISK were implemented based on openCV-Python tools [37]. In order to better display the matching results of local features of the acousto-optic images, the grayscale display mode was adopted.

### 5.3. Test and Evaluate Results

**Figure 8:**
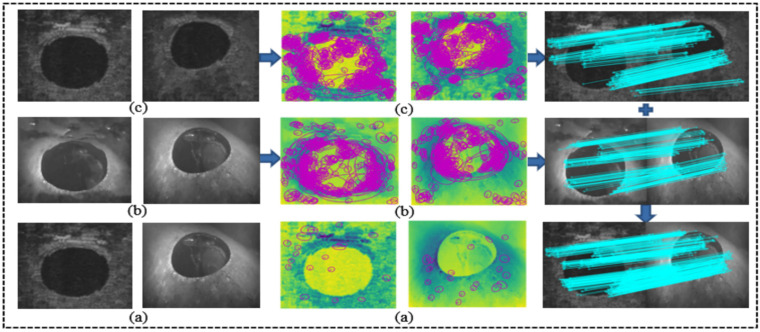
Each image sample in the experiment is tested according to the method described in Figure 8. Due to the limitations of underwater images, such as poor quality, high ambiguity, complex noise, etc., in the matching process we use the RANSAC [38] algorithm and cross-check to improve the accuracy, and the distance ratio is set to the standard value 0.8.

Due to the huge difference in imaging mechanism between the acoustic sensor and optical sensor, it is difficult to detect the feature points on the raw image pair. After preprocessing the raw image with image attribute transfer algorithm, the image difference is basically eliminated. At this time, dense feature points could be detected in both the acoustic domain and optical domain, and then they could be effectively matched in their respective domains. We aggregate the matching results of the acoustic image pair and the optical image pair, and mapped them to the raw image after the error elimination operation. We take one of these samples to specifically express the three processes of matching and visualize the detection result of feature points and feature areas. The detailed process is shown in Figure 8.

**Figure 9:**
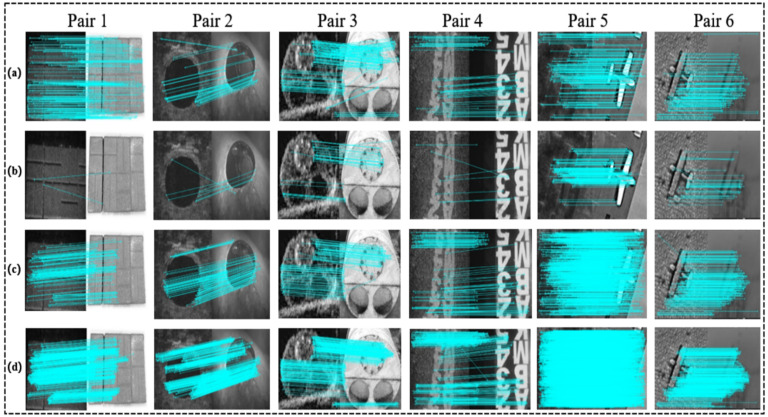
This is a schematic diagram of the matching results of the images after attribute transfer: (**a**) matching results using SIFT, (**b**) matching results using BRISK, (**c**) matching results using SURF, and (**d**) matching results using HesAffNet + HardNet.

The matching effects are shown in Figure 9, and the detailed evaluation results from image pair 1 to image pair 6 are presented in Table 1.

## 6. Discussion

As shown in Table 1, from image pair 1 to image pair 4, they prove that when the underwater images have affine transformations, the matching results obtained by our method have the highest quality. In image pair 5 and image pair 6, when there are only imaging differences, our method can also acquire the densest matching pairs.

For the underwater acousto-optic image matching task, we have solved it based on the following aspects: (1) Use the image attribute transfer method to maximize the elimination of the difference between the underwater acoustic image and optical image. (2) Introduce the advanced HardNet descriptor and use the method based on local features to match the acoustic and optical images. Experimental verification: although HardNet has not been deliberately trained on underwater sonar and optical datasets, it nonetheless shows impressive results, and could overcome the viewing angle and background changes caused by the sonar detection process. The proposed method does not make any assumptions concerning the type of input acousto-optic image, nor does it require manual intervention and preprocessing—the matching process is close to end-to-end. We have achieved a relative balance of high matching accuracy and low computational complexity. Improving the efficiency of the algorithm will be the focus of our next work.

## 7. Conclusions

A method of applying a visual attribute transfer algorithm and the learned descriptor HardNet is proposed to achieve the task of underwater acousto-optic image matching. Our method could be applied to various underwater operation scenarios and there is no need to superimpose complex sonar image processing methods. The experiment proves our proposed method could effectively solve with high accuracy and robustness the matching problem of underwater acousto-optic images.

In the future, we will focus on further expanding the number of test samples and training local feature descriptors in the underwater acousto-optic images to achieve better performance. Additionally, we plan to further optimize the preprocessing algorithm for image attribute transfer to make it lightweight, in order to better meet the real-time requirements of underwater engineering operations and to enhance the autonomy of deep submersibles.

## Figures and Tables

**Table 1:** Evaluation results of image pair 1 to image pair 6.

		Pair 1				Pair 2				Pair 3			
	Evaluation	GM	INL	MA	RT(s)	GM	INL	MA	RT(s)	GM	INL	MA	RT(s)
Methods													
Proposed + SIFT		067	036	0.5373	0.1326	068	051	0.7500	0.1207	233	145	0.6223	0.3627
Proposed + BRISK		002	001	0.5000	0.1176	013	007	0.5385	0.1396	044	033	0.7500	0.1776
Proposed + SURF		221	123	0.5566	0.2474	159	096	0.6038	0.2615	191	097	0.5078	0.2823
Proposed + HesAffNet + HardNet		842	487	0.5784	2.8483	713	413	0.5792	2.7706	422	286	0.6778	2.7195
		**Pair 4**				**Pair 5**				**Pair 6**			
	**Evaluation**	**GM**	**INL**	**MA**	**RT(s)**	**GM**	**INL**	**MA**	**RT(s)**	**GM**	**INL**	**MA**	**RT(s)**
**Methods**													
Proposed + SIFT		102	060	0.5882	0.1566	243	243	1.0000	0.1237	136	130	0.9558	0.2175
Proposed + BRISK		012	007	0.5833	0.1596	169	168	0.9941	0.1096	033	031	0.9393	0.1556
Proposed + SURF		189	087	0.4603	0.2763	1097	1096	0.9990	0.2474	214	185	0.8645	0.2503
Proposed + HesAffNet + HardNet		396	227	0.5732	2.5351	4388	4388	1.0000	2.6928	526	493	0.9373	2.5045

## Data Availability

Not applicable.
